# High-resolution data from Laser Ablation-ICP-MS and by ICP-OES analyses at the Cretaceous/Paleogene boundary section at Agost (SE Spain)

**DOI:** 10.1016/j.dib.2018.04.118

**Published:** 2018-05-03

**Authors:** Claudia Sosa-Montes de Oca, Gert J. de Lange, Francisca Martínez-Ruiz, Francisco J. Rodríguez-Tovar

**Affiliations:** a Departamento de Estratigrafía y Paleontología, Universidad de Granada, Avda. Fuentenueva s/n, 18002 Granada, Spain; b Department of Earth Sciences–Geochemistry, Utrecht University, 3584 CD Utrecht, The Netherlands; c Instituto Andaluz de Ciencias de la Tierra, IACT (CSIC-Universidad de Granada), Avda. Las Palmeras 4, 18100 Armilla, Granada, Spain

## Abstract

A high-resolution analysis of the distribution of major and trace elements across the Cretaceous/Paleogene boundary (KPgB) in the distal section of Agost (SE Spain) was performed. The KPgB sediments were drilled to recover a 22 cm-long core; the lower 5 cm corresponding to the uppermost Maastrichtian and the upper 17 cm to the lowermost Danian. The unconsolidated sediments were resin-embedded under O_2_-free conditions, cut and polished. Laser Ablation-Inductivity Coupled Plasma-Mass Spectrometry (LA-ICP-MS) analyses were conducted at 10 µm increments and a laser-beam of 80 µm. Discrete samples were taken immediately prior to the resin-embedding and analyzed by Inductivity Coupled Plasma-Optical Emission Spectroscopy (ICP-OES). Results obtained by both analytical methods (LA-ICP-MS and ICP-OES) are presented. (Further interpretations and discussion are included in Sosa-Montes de Oca et al., 2018 [6]).

**Specifications table**

**Table:** 

Subject area	*Sedimentary geochemistry*
More specific subject area	*Paleoenvironmental changes across KPgB*
Type of data	*Figures, Excel file, Table*
How data was acquired	– COMPex 102 ArF excimer laser ablation system (Lambda Physik, Göttingen, Germany) connected to an Element 2 sector field ICP-MS (Thermo Scientific, Bremen, Germany) was used for LA-ICP-MS analyses at the GML from Utrecht University (the Netherlands) – Spectro Ciros Vision ICP-OES at the Geolab from Utrecht University (The Netherlands)
Data format	*Analyzed*
Experimental factors	*Previously resin embedding processes*
Experimental features	*High resolution profiles across the KPgB sediments*
Data source location	*Agost, Alicante (Spain)*
	*Latitude: 38°27′3.31′N; Longitude: 0°-38′-9.71′′E*
Data accessibility	*Data are included in this article*

**Value of the data**

• Data show continuous high-resolution element profiles across the KPgB.
• Data reveal significant changes in elemental ratios as Ca/Al, P/Al, Sr/Al, Ti/Al, Cr/Al, Co/Al, Cu/Al, Zr/Al, Pb/Al and U/Al within the ≈2 mm-thick KPg ejecta layer.
• Data contribute to improve the characterization of major and trace element distribution. This high-resolution approach is found to as reliable tool to evaluate rapid paleoenvironmental changes associated with bio-events.

## 1 Data

The boundary between the Cretaceous and Paleogene periods has been widely investigated [1], [2], [3]. Numerous KPgB sections are known worldwide [4], the Agost site (SE, Spain) being a very well-preserved and well-exposed marine distal section [5]. This section has been profusely studied, due to its exceptionally, expansive and continuous sedimentary record, making the Agost site ideal for high-resolution analyses [6].

**Fig. 1:**
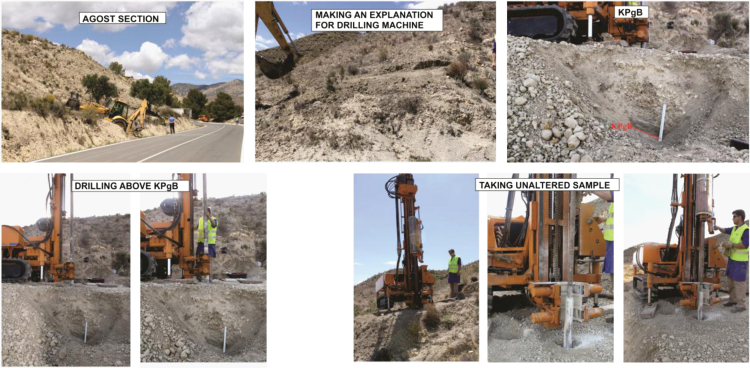
Photographs of the Agost site, during the drilling and unaltered sampling.

The KPgB was drilled using a Rolatec RL 48L drilling machine from the Center for Scientific Instrumentation (CIC), University of Granada, Spain (Fig. 1). A platform was built for the drilling machine and an unaltered core was extracted (Fig. 1). The core was sealed and stored in a cold room.

## 2 Experimental design, materials and methods

**Fig. 2:**
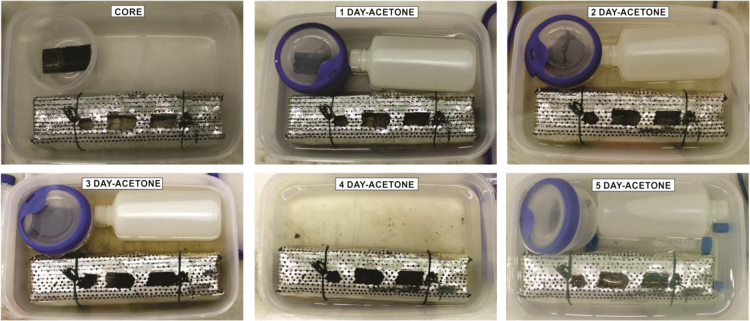
Photographs during acetone stage of resin embedding process, inside the glove box.

**Fig. 3:**
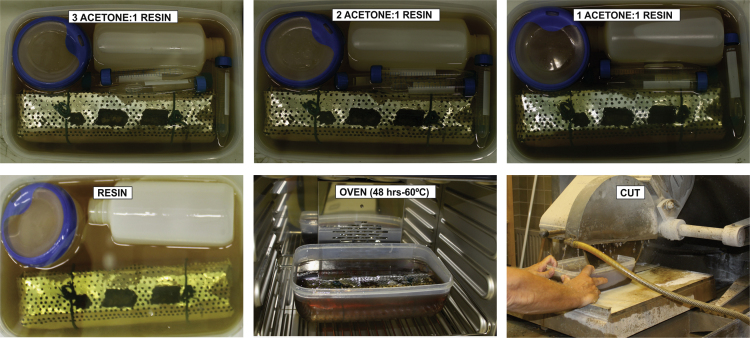
Photographs during resin stage of resin embedding process, in the oven (at 60° for 48 h) for curing and drying and while cutting the arrays for LA-ICP-MS analysis.

Discrete samples were taken for ICP-OES analysis (Table 1). Next, the core was prepared for resin embedding in order to preserve redox-sensitive elements while maintaining the material structure. All the resin embedding processes were done in an argon-filled glove box for 32 days, in two different stages: First, with acetone exchange during five days (Fig. 2), and secondly with Spurr Epoxy Resin exchange for 27 days (Fig. 3). Afterwards, the core was removed from the glove box and put into the oven for curing and drying 48 h at 60 °C. The embedded core was cut perpendicular to the bedding plane (Fig. 3), polished, and then cut to obtain 2 overlapping arrays (~5 cm each one), which were analyzed by means of a LA-ICP-MS line-scan.

**Table 1:** Table with the elemental content (major and trace) and elemental ratios, measured by ICP-OES across a 21.5 cm interval of the KPgB at the Agost section. Al, Ca, CaO, CaCO3, concentrations (%); Ca/Al and Fe/Al ratios; P/Al, Sr/Al, Ti/Al, Cr/Al, Co/Al, Cu/Al, Zr/Al and Pb/Al ratios (×10^−4^), in: i) gray calcareous marlstones and malstones from the uppermost Maastrichtian, ii) ejecta layer, iii) boundary clay layer and iv) light marly limestones from the lowermost Danian.

**Samples**	**Distance K/Pg (cm)**	**Stage**	**Lithology**	**Dilution**	**Geochemical proxies**													
					**(%)**						**(10**^**−4**^**)**							
					**Al**	**Ca**	**CaO**	**CaCO**_**3**_	**Ca/Al**	**Fe/Al**	**P/Al**	**Sr/Al**	**Ti/Al**	**Cr/Al**	**Co/Al**	**Cu/Al**	**Zr/Al**	**Pb/Al**
1	16.75		Light marly limestones	202.10	*1.68*	*35.66*	*49.94*	*89.15*	*21.17*	*0.51*	*398.75*	*346.19*	*521.87*	*20.26*	*1.96*	*5.76*	*11.62*	*0.00*
2	16.25			206.90	*1.78*	*35.61*	*49.88*	*89.03*	*20.06*	*0.51*	*397.71*	*332.79*	*518.82*	*20.17*	*1.63*	*5.11*	*11.96*	*0.00*
3	15.75			237.10	*1.70*	*35.42*	*49.60*	*88.54*	*20.81*	*0.50*	*379.76*	*338.81*	*516.70*	*19.37*	*1.67*	*4.89*	*11.73*	*0.00*
4	15.25			194.40	*1.63*	*36.21*	*50.72*	*90.53*	*22.17*	*0.50*	*383.00*	*348.24*	*504.99*	*19.15*	*1.36*	*4.37*	*11.61*	*0.00*
5	14.75			206.40	*1.91*	*35.37*	*49.54*	*88.43*	*18.57*	*0.50*	*390.80*	*307.70*	*511.28*	*19.24*	*1.75*	*4.65*	*12.30*	*0.00*
6	14.25			193.00	*2.23*	*33.87*	*47.44*	*84.68*	*15.22*	*0.47*	*355.15*	*270.94*	*508.91*	*19.60*	*1.21*	*4.13*	*11.58*	*0.00*
7	13.75			236.00	*2.57*	*31.91*	*44.69*	*79.78*	*12.41*	*0.45*	*343.84*	*237.45*	*524.10*	*19.75*	*1.13*	*3.62*	*11.77*	*0.00*
8	13.25			200.60	*2.79*	*31.10*	*43.56*	*77.75*	*11.15*	*0.44*	*338.31*	*221.49*	*508.37*	*19.91*	*1.04*	*3.55*	*11.52*	*0.00*
9	12.75			244.60	*2.84*	*30.22*	*42.33*	*75.56*	*10.66*	*0.44*	*335.90*	*218.27*	*510.53*	*19.99*	*1.02*	*3.71*	*12.18*	*0.00*
10	12.25			226.40	*2.87*	*29.96*	*41.96*	*74.89*	*10.45*	*0.43*	*328.96*	*214.86*	*504.26*	*19.74*	*1.03*	*3.65*	*11.39*	*0.00*
11	11.75			193.20	*2.70*	*30.66*	*42.94*	*76.65*	*11.34*	*0.43*	*319.30*	*227.52*	*503.14*	*19.56*	*0.99*	*3.63*	*11.38*	*0.00*
12	11.25			202.10	*2.58*	*31.93*	*44.73*	*79.84*	*12.37*	*0.44*	*324.56*	*240.72*	*501.64*	*19.28*	*1.11*	*3.63*	*11.41*	*0.00*
13	10.75			243.00	*2.68*	*30.87*	*43.23*	*77.16*	*11.52*	*0.44*	*319.09*	*238.43*	*504.08*	*19.32*	*1.27*	*4.18*	*11.97*	*0.00*
14	10.25			232.80	*2.78*	*29.90*	*41.87*	*74.74*	*10.74*	*0.44*	*313.01*	*230.64*	*505.10*	*19.47*	*1.20*	*3.82*	*11.59*	*0.00*
15	9.75			239.30	*3.13*	*28.75*	*40.27*	*71.88*	*9.18*	*0.43*	*310.19*	*207.23*	*511.91*	*19.78*	*1.20*	*3.55*	*11.45*	*0.00*
16	9.25			202.30	*2.91*	*30.42*	*42.60*	*76.04*	*10.47*	*0.43*	*305.80*	*229.76*	*514.81*	*19.94*	*1.26*	*3.89*	*11.34*	*0.00*
17	8.75			223.10	*2.87*	*29.50*	*41.31*	*73.75*	*10.28*	*0.44*	*309.70*	*228.52*	*514.59*	*19.60*	*1.59*	*3.98*	*11.09*	*0.00*
18	8.25			223.20	*2.50*	*30.86*	*43.22*	*77.14*	*12.33*	*0.48*	*292.41*	*265.57*	*518.21*	*19.17*	*1.95*	*4.29*	*11.95*	*0.00*
19	7.75			238.10	*2.23*	*31.86*	*44.62*	*79.65*	*14.32*	*0.47*	*263.61*	*299.56*	*527.33*	*18.11*	*2.18*	*4.49*	*12.63*	*0.00*
20	7.25			233.70	*2.17*	*31.41*	*43.99*	*78.52*	*14.49*	*0.44*	*243.38*	*307.65*	*530.01*	*17.98*	*1.62*	*4.32*	*13.70*	*0.00*
21	6.75			229.30	*2.18*	*31.09*	*43.55*	*77.74*	*14.24*	*0.45*	*217.36*	*313.54*	*529.64*	*17.80*	*2.01*	*4.51*	*14.21*	*0.00*
22	6.25	Danian		236.10	*2.49*	*30.02*	*42.05*	*75.06*	*12.06*	*0.44*	*238.68*	*311.23*	*521.08*	*18.24*	*9.84*	*4.99*	*12.84*	*0.00*
23	5.75			257.90	*2.90*	*30.07*	*42.11*	*75.17*	*10.37*	*0.45*	*276.10*	*296.11*	*498.49*	*18.78*	*6.02*	*5.24*	*10.63*	*0.00*
24	5.25			233.00	*2.62*	*31.35*	*43.91*	*78.38*	*11.98*	*0.50*	*227.18*	*368.52*	*486.07*	*19.23*	*6.64*	*5.93*	*10.33*	*0.00*
25	4.875		Boundary clay layer	227.50	*6.58*	*16.92*	*23.70*	*42.31*	*2.57*	*0.42*	*140.80*	*124.41*	*481.49*	*19.99*	*3.57*	*5.75*	*10.57*	*4.69*
26	4.625			218.30	*6.51*	*17.16*	*24.04*	*42.90*	*2.64*	*0.41*	*125.55*	*117.04*	*477.42*	*19.42*	*2.22*	*5.50*	*10.67*	*4.65*
27	4.375			208.60	*7.53*	*13.12*	*18.37*	*32.79*	*1.74*	*0.41*	*112.49*	*95.77*	*472.94*	*19.71*	*2.06*	*5.29*	*11.06*	*5.42*
28	4.125			201.00	*7.75*	*12.07*	*16.91*	*30.18*	*1.56*	*0.42*	*100.11*	*91.93*	*469.09*	*19.97*	*1.98*	*5.46*	*11.08*	*5.77*
29	3.875			207.70	*7.75*	*11.98*	*16.78*	*29.95*	*1.55*	*0.40*	*92.62*	*92.97*	*450.06*	*20.89*	*1.98*	*6.21*	*11.41*	*5.40*
30	3.625			230.50	*8.15*	*10.37*	*14.53*	*25.94*	*1.27*	*0.40*	*90.83*	*87.18*	*460.77*	*22.66*	*1.92*	*7.46*	*11.85*	*5.55*
31	3.375			241.80	*7.84*	*10.76*	*15.07*	*26.89*	*1.37*	*0.40*	*90.90*	*88.38*	*472.00*	*21.98*	*2.14*	*6.15*	*11.65*	*5.10*
32	3.125			211.80	*7.66*	*11.50*	*16.11*	*28.76*	*1.50*	*0.41*	*90.49*	*93.11*	*468.99*	*22.04*	*2.38*	*5.95*	*11.93*	*5.77*
33	2.875			197.60	*8.05*	*10.02*	*14.03*	*25.05*	*1.24*	*0.43*	*80.61*	*89.52*	*461.46*	*23.76*	*1.76*	*6.60*	*11.59*	*6.32*
34	2.625			222.10	*8.69*	*8.40*	*11.77*	*21.01*	*0.97*	*0.43*	*69.60*	*84.55*	*473.17*	*25.35*	*1.57*	*6.05*	*11.58*	*6.09*
35	2.375			250.80	*8.02*	*10.52*	*14.73*	*26.29*	*1.31*	*0.45*	*81.34*	*93.84*	*490.22*	*26.60*	*1.84*	*7.02*	*11.94*	*5.41*
36	2.125			212.00	*7.66*	*9.90*	*13.87*	*24.76*	*1.29*	*0.49*	*87.75*	*94.61*	*488.33*	*26.30*	*2.01*	*9.06*	*12.17*	*6.38*
37	1.875			234.80	*7.94*	*9.42*	*13.20*	*23.56*	*1.19*	*0.49*	*87.02*	*93.77*	*500.66*	*27.66*	*2.21*	*9.82*	*12.18*	*6.35*
38	1.625			223.20	*7.95*	*9.08*	*12.72*	*22.70*	*1.14*	*0.56*	*86.63*	*94.58*	*494.52*	*28.22*	*2.56*	*12.27*	*12.72*	*7.27*
39	1.375			238.20	*7.85*	*8.17*	*11.45*	*20.43*	*1.04*	*0.62*	*79.13*	*94.60*	*474.29*	*28.67*	*2.72*	*11.99*	*13.11*	*6.89*
40	1.125			218.60	*8.06*	*7.16*	*10.03*	*17.90*	*0.89*	*0.69*	*79.40*	*91.32*	*476.25*	*29.63*	*2.94*	*13.27*	*14.22*	*7.96*
41	0.875			223.30	*7.83*	*8.24*	*11.53*	*20.59*	*1.05*	*0.66*	*89.23*	*94.34*	*554.95*	*33.19*	*2.70*	*14.13*	*14.70*	*8.32*
42	0.625			234.60	*7.32*	*9.22*	*12.92*	*23.06*	*1.26*	*0.76*	*85.71*	*105.52*	*857.39*	*48.29*	*3.10*	*17.82*	*17.15*	*10.46*
43	0.35	KPgB	Ejecta layer	254.30	*5.47*	*17.43*	*24.41*	*43.58*	*3.19*	*0.75*	*79.67*	*150.69*	*995.28*	*49.95*	*3.52*	*17.10*	*16.78*	*8.78*
44	0.15			236.40	*4.57*	*23.50*	*32.92*	*58.76*	*5.14*	*0.52*	*93.61*	*190.95*	*673.70*	*30.30*	*2.70*	*8.91*	*12.61*	*4.33*
45	0			242.20	*4.46*	*23.87*	*33.43*	*59.66*	*5.35*	*0.59*	*96.98*	*198.41*	*960.64*	*41.60*	*3.85*	*18.71*	*14.56*	*7.01*
46	−0.25	Maastrichtian	Calcareous marlstones and marlstones	203.50	*3.98*	*25.86*	*36.22*	*64.65*	*6.50*	*0.41*	*116.58*	*224.05*	*505.28*	*20.89*	*1.85*	*7.99*	*10.71*	*2.91*
47	−0.75			224.00	*3.73*	*26.82*	*37.56*	*67.04*	*7.19*	*0.37*	*127.08*	*239.79*	*455.49*	*17.90*	*1.46*	*6.24*	*10.55*	*0.22*
48	−1.25			206.80	*4.08*	*25.47*	*35.67*	*63.66*	*6.24*	*0.40*	*111.37*	*217.01*	*559.27*	*23.63*	*1.46*	*6.90*	*10.99*	*3.25*
49	−1.75			224.60	*3.12*	*29.34*	*41.10*	*73.36*	*9.42*	*0.36*	*136.43*	*290.95*	*432.65*	*15.96*	*1.56*	*4.96*	*10.41*	*0.00*
50	−2.25			217.80	*3.08*	*29.14*	*40.81*	*72.85*	*9.47*	*0.36*	*136.55*	*293.20*	*427.42*	*15.99*	*1.47*	*5.19*	*10.41*	*0.00*
51	−2.75			210.50	*2.88*	*30.93*	*43.32*	*77.33*	*10.76*	*0.37*	*144.07*	*318.96*	*458.63*	*17.01*	*1.25*	*5.07*	*10.36*	*0.00*
52	−3.25			193.50	*2.76*	*31.56*	*44.20*	*78.89*	*11.44*	*0.38*	*148.41*	*338.23*	*464.05*	*17.32*	*1.41*	*4.97*	*10.67*	*0.00*
53	−3.75			196.10	*2.77*	*31.76*	*44.48*	*79.39*	*11.46*	*0.38*	*144.27*	*340.32*	*459.90*	*16.84*	*1.24*	*5.67*	*10.20*	*0.00*
54	−4.25			211.50	*2.61*	*31.73*	*44.44*	*79.33*	*12.14*	*0.38*	*153.29*	*359.35*	*466.51*	*16.88*	*1.38*	*5.09*	*10.65*	*0.00*
55	−4.75			204.40	*2.89*	*29.70*	*41.60*	*74.26*	*10.27*	*0.40*	*160.49*	*319.98*	*509.64*	*19.94*	*1.28*	*5.16*	*10.86*	*0.00*

Here we present the geochemical data obtained using both techniques across the KPgB, including the ejecta layer. In the LA-ICP-MS profiles, 4114 data points were obtained in a 9 mm studied interval specifically, 544 data points in the gray calcareous marlstones and marlstones from the uppermost Maastrichtian, and 3570 data points from the lowermost Danian sediments. Among the latter, 255 data points were taken in the ejecta layer, 1827 data points in the boundary clay layer and 1488 data points in the light marly limestones (Excel file 1 from Supplementary material). In turn, the ICP-OES profiles include only 31 data points in a 21.50 cm studied interval, four of which correspond to the gray calcareous marlstones and marlstones from the uppermost Maastrichtian and 27 to the lowermost Danian sediments. Of these 27, three data points were taken in the ejecta layer, 16 in the boundary clay layer and 8 data points in the light marly limestones (Table 1).

The counts obtained through LA-ICP-MS analysis were interpreted, corrected for background noise, and calibrated. First, the relative ionization factors of the NIST610 standards were calculated. To this end, a NIST610 standard was tested between each sample line-scan analysis and the measurement counts are associated with concentrations by using ratios relative to Al (ppm/counts ratio relative to Al=1) (Excel file 2 from Supplementary material). Then the LA-ICP-MS line-scans obtained for the different isotopes were also: i) corrected for background, subtracting the mean background values obtained from the average intensities of a ~30 s interval before starting the laser ablation measurement; ii) the background-corrected analyte intensities were corrected for the relative sensitivity of the specific isotope calculated by measuring an external standard (using the NIST610 values previously calculated) [7]; iii) the natural abundance of each isotope was corrected [8]; iv) lastly, data were reported as ratios of an internal standard (in this case Al) because the yield of ablated material varies during LA-ICP-MS.

The profiles are presented as (log-) ratios, since they are statistically more informative//precise than normal ratios; in addition, on μm- to mm-scales no internal standard with a known concentration is available during LA-ICP-MS line-scanning of natural samples (Excel file 3 from Supplementary material).

The ICP-OES data are furthermore used as an extra calibration step by means of simple regression, so that both data sets can be compared for the same interval.

## Appendix A Supplementary material

**Excel file 1**. Elemental isotopic ratio data, as measured by LA-ICP-MS in a 9-cm interval across the KPgB at the Agost site. Moving average (in a 5-and 10- intervals) of Ca/Al, P/Al, Sr/Al, Ti/Al, Cr/Al, Co/Al, Cu/Al, Zr/Al, Pb/Al and U/Al ratios; in: i) gray calcareous marlstones and malstones from the uppermost Maastrichtian, ii) ejecta layer, iii) boundary clay layer, and iv) light marly limestones from the lowermost Danian.

**Excel file 2.** Relative ionization factors of the NIST610 standards. In this file, the NIST610 measurement counts are basically associated to their concentrations (which are known), making ratios relative to Al.

**Excel file 3.** Example file of LA-ICP-MS line scan analysis after data treatment.
