# Supplementary material for: High-resolution data from Laser Ablation-ICP-MS and by ICP-OES analyses at the Cretaceous/Paleogene boundary section at Agost (SE Spain)
Source: Data Brief. 2018 May 3;18:1900–6. doi: 10.1016/j.dib.2018.04.118 (PMC5998653; doi:10.1016/j.dib.2018.04.118)
Supplement: Supplementary file 1 — Supplementary material [file mmc1.pdf]

Universidad de Granada
DEPARTAMENTO DE ESTRATIGRAFÍA
Y PALEONTOLOGÍA

AUTHOR DECLARATION

April, 2018

We wish to confirm that there are no known conflicts of interest associated with this publication and there has been no significant financial support for this work that could have influenced its outcome.

We confirm that the manuscript has been read and approved by all named authors and that there are no other persons who satisfied the criteria for authorship but are not listed. We further confirm that the order of authors listed in the manuscript has been approved by all of us.

We confirm that we have given due consideration to the protection of intellectual property associated with this work and that there are no impediments to publication, including the timing of publication, with respect to intellectual property. In so doing we confirm that we have followed the regulations of our institutions concerning intellectual property.

We understand that the Corresponding Author is the sole contact for the Editorial process (including Editorial Manager and direct communications with the office). She is responsible for communicating with the other authors about progress, submissions of revisions and final approval of proofs. We confirm that we have provided a current, correct email address which is accessible by the Corresponding Author and which has been configured to accept email from (csosa@ugr.es)

On behalf of all co-authors, sincerely,

Claudia

Claudia Sosa Montes de Oca
Dpto de Estratigrafía y Paleontología
Universidad de Granada, Spain
Campus Fuente Nueva S/N, 18002
Tel.: (34) 958 23 00 00 ext.: 190029
e-mail: csosa@ugr.es; claudia.sosa@csic.es

FACULTAD DE CIENCIAS
CAMPUS FUENTENUEVA S/N
18002 GRANADA. ESPAÑA
TEL: (34) 958 23 00 00
ext. 190029
FAX: (34) 958 55 26 20
